# Uncovering Hypercalcemia of Malignancy Following Parathyroidectomy for Hyperparathyroidism

**DOI:** 10.1210/jcemcr/luae203

**Published:** 2024-11-14

**Authors:** Lutfor Nessa, Joud Enabi, Samhitha Gonuguntla, Bosky Modi, Maida Faheem, Anand Reddy

**Affiliations:** Department of Internal Medicine, Texas Tech University Health Sciences Center Permian Basin, Odessa, TX 79763, USA; Department of Internal Medicine, Texas Tech University Health Sciences Center Permian Basin, Odessa, TX 79763, USA; Department of Internal Medicine, Texas Tech University Health Sciences Center Permian Basin, Odessa, TX 79763, USA; Department of Internal Medicine, Texas Tech University Health Sciences Center Permian Basin, Odessa, TX 79763, USA; Department of Internal Medicine, Texas Tech University Health Sciences Center Permian Basin, Odessa, TX 79763, USA; Department of Nephrology, Permian Basin Kidney Center, Odessa, TX 79761, USA

**Keywords:** hypercalcemia, malignancy, diffuse large B-cell carcinoma, hyperparathyroidism

## Abstract

Hypercalcemia is a common complication of malignancy, often attributed to elevated PTH-related protein levels mimicking the effects of PTH and promoting bone resorption. We present the case of a 67-year-old Hispanic female with a history of hypertension, type 2 diabetes mellitus, hyperlipidemia, and chronic kidney disease, who initially underwent parathyroidectomy for suspected primary hyperparathyroidism resulting from persistent hypercalcemia. Despite surgery, the patient continued to experience hypercalcemia and was subsequently diagnosed with diffuse large B-cell lymphoma with hypercalcemia as a rare but life-threatening complication. This case highlights the importance of considering malignancy as a potential cause of hypercalcemia, particularly in the context of hematologic malignancies such as diffuse large B-cell lymphoma. Prompt recognition and management are crucial to prevent severe complications associated with hypercalcemia. A multidisciplinary approach involving oncologists, endocrinologists, and supportive care teams is essential for effective management in such cases.

## Introduction

Calcium, a vital extracellular cation, governs crucial structural and enzymatic functions [1], regulated meticulously by calcitonin, vitamin D, and PTH. Hypercalcemia, marked by serum calcium levels exceeding 10.50 mg/dL (2.62 mmol/L), ranges from mild to severe, with symptoms spanning nausea, vomiting, dehydration, and confusion to coma [2]. Understanding the mechanisms of hypercalcemia may help to delineate the primary underlying disorder responsible for the elevated calcium levels. Predominantly stemming from primary hyperparathyroidism and malignancy, hypercalcemia also manifests in granulomatous disorders and medication-induced cases [2]. It is also linked to various hematological and solid cancers, including multiple myeloma, non-Hodgkin lymphoma, leukemias, renal and breast carcinomas, and squamous cell carcinomas, with osteolytic-related hypercalcemia and hormone-mediated hypercalcemia contributing to its development [3].

Diffuse large B-cell lymphoma (DLBCL) is a prevalent variety of non-Hodgkin lymphoma, accounting for 25% of cases and 7 cases per 100 000 people annually in the United States [4]. DLBCL exhibits substantial clinical heterogeneity, despite molecular classification efforts focusing on predicting therapeutic outcomes. Extranodal involvement is more prevalent in DLBCL, with approximately 40% of patients with localized disease exhibiting extranodal involvement, most frequently in the gastrointestinal system [4]. Despite DLBCL's prevalence, the occurrence of hypercalcemia within this subtype remains unclear [5]. Although hypercalcemia correlates with poor cancer prognosis, its direct impact remains elusive. Various treatment options are available depending on the underlying cause, including parathyroidectomy for primary hyperparathyroidism and hydration with IV bisphosphonates for severe hypercalcemia [2]. Given its prognostic significance, timely identification and treatment of hypercalcemia's root cause are imperative. In this report, we present a case where hypercalcemia initially prompted parathyroidectomy but was subsequently linked to DLBCL.

## Case Presentation

We present a 67-year-old Hispanic female with a medical history significant for hypertension, type 2 diabetes mellitus (HbA1c 6.8%; 74.28 mmol/mol), hyperlipidemia, chronic kidney disease stage 3a, and left ureteral stent placement in 2018 for a left ureteral calculus. During a routine follow-up with her primary care provider, she was noted to have persistently elevated serum calcium levels.

## Diagnostic Assessment

Further evaluation revealed elevated intact PTH at 110 pg/mL (11.68 pmol/L) (normal reference range, 10-65 pg/mL; 1.06-6.90 pmol/L), calcium at 12.30 mg/dL (3.06 mmol/L) (normal reference range, 8.50-10.20 mg/dL; 2.13-2.55 mmol/L), serum albumin at 3.70 g/dL (37 g/L) (normal reference range, 3.50-5.0 g/dL; 35-50 g/L), vitamin D at 12.30 ng/mL (30.74 nmol/L) (normal reference range, 20-40 ng/mL; 49.92 to 99.84 nmol/L), Serum phosphate level at 3.50 mg/dL (0.04 mmol/L) (normal reference range, 2.50-4.50 mg/dL; 0.81-1.45 mmol/L) and renal function test blood urea nitrogen at 49 mg/dL (0.82 mmol/L) (normal reference range, 7-20 mg/dL; 2.50-7.10 mmol/L) and creatinine at 2.70 mg/dL (23.90 µmol/L) (normal reference range, 0.50-1.10 mg/dL; 44-97 µmol/L). Previous suspicions of a parathyroid adenoma led to nuclear medicine parathyroid imaging (single-photon emission computed tomography-computed tomography), which did not reveal an adenoma. Because of persistent hypercalcemia, she underwent parathyroidectomy, with pathology confirming a parathyroid adenoma of the right lower gland. Laboratory investigations recorded within a few days after the parathyroidectomy revealed PTH at 5.60 pg/mL (5.96 × 10^−4^ pmol/L) (normal reference range, 10-65 pg/mL; 1.06-6.90 pmol/L), calcium at 11.80 mg/dL (2.94 mmol/L) (normal reference range, 8.50-10.20 mg/dL; 2.13-2.55 mmol/L), and serum phosphate at 3.50 mg/dL (1.13 mmol/L) (normal reference range 2.50-4.50 mg/dL; 0.81-1.45 mmol/L). However, following the parathyroidectomy, the patient experienced intermittent abdominal pain accompanied by nausea, with normal abdominal examination findings. Laboratory workup was needed, which showed elevated serum calcium at 14.10 mg/dL (3.51 mmol/L) (normal reference range, 8.50-10.20 mg/dL; 2.13-2.55 mmol/L) and elevated PTH-related protein (PTHrP) at 4.30 pmol/L (452.63 pmol/L) (normal reference range <2 pg/mL; <2.12 pmol/L). Subsequent evaluation demonstrated negative β human chorionic gonadotropin, no signs of infection, and elevated urine kappa and lambda with a negative M-spike on serum protein electrophoresis. Serum PTH was high-normal preparathyroidectomy and low postparathyroidectomy. 1,25-dihydroxyvitamin D was normal, and repeat PTHrP was elevated at 6.80 pmol/L (64 600 pg/mL) (normal reference range, <2 pg/mL; <2.12 pmol/L). Computed tomography of the abdomen showed pelvic organ thickening, intra-abdominal fat edema with a nodular omental pattern, and moderate ascites. Ultrasonography of the pelvis indicated simple anechoic fluid in the endometrial cavity. Elevated tumor markers, including CA 125 at 2310.0 U/mL (2.31 kU/L) (normal level, <38.10 U/mL; <38.10 kU/L) and carcinoembryonic antigen at 4.30 ng/mL (4.30 µg/L) (normal level, <3 ng/mL; <3 µg/L), raised suspicion for gynecological malignancy, for which obstetrics and gynecology and oncology were consulted.

## Treatment

Zoledronic acid was given for the elevated serum calcium, but it showed no significant improvement in serum calcium levels. Despite elevated CA 125 levels, gynecological malignancy was not confirmed on further evaluation. No malignant cells were found in the ascitic fluid from paracentesis, and the Papanicolaou smear of the cervix showed a high-grade squamous intraepithelial lesion. An infrared-guided peritoneal biopsy revealed atypical B-cell proliferation, and subsequent investigations, including nuclear medicine bone imaging and percutaneous bone marrow biopsy, were negative for metastatic disease or osseous malignancies. In the third week, the patient experienced worsening abdominal pain, nausea, and vomiting. Repeat computed tomography of the abdomen revealed severe cervical and vaginal wall thickening and an exophytic fibroid from the fundal region, prompting a loop electrosurgical excision procedure cone cervical biopsy, which revealed diffuse large B-cell lymphoma (germinal center type). A computed tomography-guided bone marrow biopsy confirmed the diagnosis of diffuse large B-cell lymphoma. The patient was initiated on the Rituximab-Cyclophosphamide, Doxorubicin, Vincristine, and Prednisone regimen for lymphoma treatment.

## Outcome and Follow-up

After Rituximab-Cyclophosphamide, Doxorubicin, Vincristine, and Prednisone treatment, the patient reported improvement in abdominal pain and routine laboratory findings showed improvement in calcium levels at 8.30 mg/dL (2.07 mmol/L) (normal reference range, 8.50-10.20 mg/dL; 2.13-2.55 mmol/L) and PTHrP at 2.50 pmol/L (35.25 pg/mL) (normal reference range, <2 pg/mL; <2.12 pmol/L).

## Discussion

Hypercalcemia is a well-documented complication of malignancy, occurring in various malignant and nonmalignant conditions. Elevated PTHrP levels, often associated with malignancies, can mimic the effects of PTH, leading to bone resorption and subsequent hypercalcemia [6]. Although hypercalcemia is more commonly observed in solid tumor malignancies, hematologic malignancies such as DLBCL can also present this complication, albeit less frequently. In hematologic malignancies, hypercalcemia may be attributed to tumor cell production of excessive vitamin D [7].

Only a few instances have been reported of B-cell lymphomas causing hypercalcemia induced by PTHrP. Although hypercalcemia is frequently observed in adult T-cell lymphomas/leukemia and solid tumors because of excessive production of PTHrP, <10% of patients with non-Hodgkins B-cell lymphomas experience hypercalcemia. Hypercalcemia in B-cell lymphomas is usually caused by mechanisms unrelated to PTHrP [8]. The pathophysiology behind hypercalcemia in hematologic malignancies has multiple explanations [8]. Most case studies reporting B-cell lymphomas with hypercalcemia secondary to PTHrP hypersecretion involve high-grade lymphomas. Several case reports have demonstrated hypercalcemia in B-cell lymphoma is a poor prognostic factor [9]. A study showed that the median survival time after developing hypercalcemia was 9 months in the 8 patients he reported [10]. Humoral PTHrP from metastatic malignancies can lead to lethal hypercalcemia if not diagnosed and treated accordingly [11]. Severe hypercalcemia can be fatal and can present as coma, epileptic seizures, acute heart failure, and acute renal failure. Therefore, fluid resuscitation and the use of medications to lower serum calcium concentration, including calcitonin, denosumab, and zoledronic acid are commonly used for the treatment of those cases [12].

Managing hypercalcemia in DLBCL patients presents challenges and requires a multidisciplinary approach involving oncologists, endocrinologists, and supportive care teams. Bisphosphonates are commonly used to inhibit bone resorption and lower serum calcium levels in cancer patients, including those with DLBCL [12]. Additionally, chemotherapy targeting the underlying malignancy can help reduce PTHrP levels and alleviate hypercalcemia.

It's important to note that clinicians may initially attribute hypercalcemia to hyperparathyroidism and overlook underlying malignancies. This case underscores the necessity of considering all potential causes of hypercalcemia, as demonstrated by the coexistence of hyperparathyroidism and DLBCL with hypercalcemic crisis as the initial presentation. The reported prevalence of hypercalcemia at diagnosis in non-Hodgkin lymphoma ranges from 1.30% to 7.40%, emphasizing its significance as a potential complication in hematologic malignancies [5]. In conclusion, this case highlights the diagnostic complexity of hypercalcemia and the importance of considering malignancies as potential underlying causes. Despite initial attribution to primary hyperparathyroidism, persistent hypercalcemia postparathyroidectomy unveiled DLBCL. Prompt recognition and management of DLBCL-induced hypercalcemia are crucial for optimizing patient outcomes. This underscores the necessity for a comprehensive approach to hypercalcemia evaluation, ensuring timely diagnosis and tailored treatment strategies.

## Learning Points

- Hypercalcemia can signal malignancy, necessitating thorough evaluation even when primary hyperparathyroidism is suspected.
- Diagnostic complexity underscores the need for considering various etiologies in hypercalcemia cases.
- Multidisciplinary collaboration optimizes management, especially in hypercalcemia associated with malignancy.
- Hypercalcemia in B-cell lymphomas correlates with poorer outcomes, emphasizing the urgency of intervention.
- Treatment strategies encompass bisphosphonates, fluid management, and targeted chemotherapy for effective hypercalcemia control.

## Contributors

All authors made individual contributions to authorship. L.N. and S.G. were involved in writing the introduction and case presentation. J.E. and B.M. were involved in writing case discussion. M.F. was involved in writing the conclusion. Abstract, learning points, and proofreading was done by B.M., and A.R. helped in proofreading and intellectual content.

## Data Availability

Data sharing is not applicable to this article as no data sets were generated or analyzed during the current study.

## Abbreviations

DLBCL: diffuse large B-cell lymphoma
PTHrP: PTH-related protein
